# A lipase from *Lacticaseibacillus rhamnosus* IDCC 3201 with thermostability and pH resistance for use as a detergent additive

**DOI:** 10.1007/s00253-024-13185-4

**Published:** 2024-06-06

**Authors:** Mi Dan Kang, Go Eun Choi, Jeong Hwa Jang, Sung-Chul Hong, Hee-Soo Park, Dong Hyun Kim, Won Chan Kim, Natasha P. Murphy, Young Hoon Jung

**Affiliations:** 1 School of Food Science and Biotechnology, Kyungpook National University, Daegu, 41566 Republic of Korea; 2 Department of Food Science and Biotechnology, Kunsan National University, Gunsan, 54150 Republic of Korea; 3 Department of Applied Biosciences, Department of Integrative Biology, Kyungpook National University, Daegu, 41566 Republic of Korea; 4 Renewable Resources and Enabling Sciences Center, National Renewable Energy Laboratory, Golden, CO 80401 USA

**Keywords:** Lipase, Recombinant enzyme, Detergent additive, Thermostability, pH resistance

## Abstract

**Abstract:**

Lipases are important biocatalysts and ubiquitous in plants, animals, and microorganisms. The high growth rates of microorganisms with low production costs have enabled the wide application of microbial lipases in detergent, food, and cosmetic industries. Herein, a novel lipase from *Lacticaseibacillus rhamnosus* IDCC 3201 (Lac-Rh) was isolated and its activity analyzed under a range of reaction conditions to evaluate its potential industrial application. The isolated Lac-Rh showed a molecular weight of 24 kDa and a maximum activity of 3438.5 ± 1.8 U/mg protein at 60 °C and pH 8. Additionally, Lac-Rh retained activity in alkaline conditions and in 10% v/v concentrations of organic solvents, including glycerol and acetone. Interestingly, after pre-incubation in the presence of multiple commercial detergents, Lac-Rh maintained over 80% of its activity and the stains from cotton were successfully removed under a simulated laundry  setting. Overall, the purified lipase from *L. rhamnosus* IDCC 3201 has potential for use as a detergent in industrial applications.

**Key points:**

• *A novel lipase (Lac-Rh) was isolated from Lacticaseibacillus rhamnosus IDCC 3201*

• *Purified Lac-Rh exhibited its highest activity at a temperature of 60 °C and a pH of 8, respectively*

• *Lac-Rh remains stable in commercial laundry detergent and enhances washing performance*

**Supplementary Information:**

The online version contains supplementary material available at 10.1007/s00253-024-13185-4.

## Introduction

Triacylglycerol hydrolases, or lipases (EC 3.1.1.3), are an essential class of enzymes which can catalyze a variety of processes, such as esterification, interesterification, transesterification, and hydrolysis of carboxylic ester bonds (Chandra et al. 2020; Hemlata et al. 2016; Khan et al. 2023; Rathi et al. 2001; Rmili et al. 2019; Sharma et al. 2017). They are readily obtainable from various sources such as plants, animals, and microorganisms (Hemlata et al. 2016; Khan et al. 2023; Tang et al. 2017; Ruiz-Villafán et al. 2023; Thakur et al. 2023; Zhao et al. 2021) and are renowned for their versatility in utilizing a wide array of substrates and for desirable biocatalytic properties, including high stability in organic solvents and detergents. Additionally, microbial lipase production is widely established at scale, enabled by rapid growth on affordable media (Rmili et al. 2019; Sharma et al. 2017; Zhao et al. 2021; Liu and Kokare 2023; Salgado et al. 2022).

While proteases remain the most commonly used enzymes for cleaning applications, lipases have garnered significant interest and application by detergent manufacturers (Gurkok 2019; Jurado et al. 2011). In 13 billion tons of detergents manufactured worldwide each year, around 1000 tons of lipases are included (Lailaja and Chandrasekaran 2013; Chauhan et al. 2013; Saraswat et al. 2017). The addition of lipase to detergents has several environmental benefits, including reduced energy and water consumption and decreased use of synthetic detergents (Zafar et al. 2022). Lipases enable effective grease and oil stain removal from fabrics, providing enhanced cleaning power that eliminates repeated washing (Saraswat et al. 2017; Mander et al. 2012; Zarinviarsagh et al. 2017). The first lipolytic enzyme to be used in the detergent industry on a commercial scale was Lipolase® (Dab et al. 2023; Nerurkar et al. 2013). Several other microbial lipases have been successfully integrated into detergent formulations to enhance their cleaning capabilities including Lipomax® and Lumafast® (Dab et al. 2023; Nerurkar et al. 2013; Al-Ghanayem and Joseph 2020; Grbavčić et al. 2011; Hemachander and Puvanakrishnan 2000). Meanwhile, lipases used in the laundry detergent industry require stability in detergent formulations (Hemlata et al. 2016; Gurkok 2019; Dab et al. 2023). However, a number of chemicals included in detergents such as metal ions, corrosion inhibitors, optical brighteners, bleaching agents, oxidizing agents, foam regulators, and surfactants might reduce lipase activity (Gurkok 2019; Grbavčić et al. 2011; Fulton et al. 2015; Naganthran et al. 2017). Therefore, novel microbial lipases that retain activity and stability in the presence of additives found in commercially available detergents as well as under relevant temperature and pH conditions are required.

*Lacticaseibacillus rhamnosus* is a promising candidate for the microbial production of lipases because of its established application in large-scale microbial industrial production processes. It is a “generally recognized as safe” lactic acid bacterium; hence, it is already widely produced as a probiotic in foodstuff (Saraswat et al. 2017; Chae et al. 2022). Herein, we identified a new lipase from a strain of *L. rhamnosus* IDCC 3201. We demonstrate the successful recombinant production and purification of the lipase (Lac-Rh) and describe the biochemical characterization across a range of pH, temperature, solvent, and metal ion conditions. Finally, to examine the applicability of Lac-Rh in the detergent industry, we evaluated its effectiveness in removing stains from fabrics in the presence of detergents.

## Materials and methods

### Cloning and expression of Lac-Rh

*L. rhamnosus* IDCC 3201 was obtained from Ildong Bioscience (Pyeongtaek-si, Gyeonggi-do, Korea) and was cultured as a facultative anaerobe in MRS (BD Difco, Detroit, MI, USA) medium at 37 °C overnight. Their genomic DNA was extracted using a genomic DNA purification kit (Wizard Genomic DNA Purification Kit). A template for amplification was created by using a designed forward primer (5′-CTCGGCGATTCATTGACTTATGGCG-3′) and a reverse primer (5′-ATAACCGGCATTATTAGGGTGAAAATGATC-3′). The detailed process of PCR for cloning is as follows: an initial denaturation step at 95 °C for 2 min, denaturation at 95 °C for 30 s, annealing at 54.7 °C for 60 s, and elongation at 72 °C for 1 min, repeated for 30 cycles, with a final elongation step at 72 °C for 5 min. Afterwards, it was confirmed using a 1.5% (w/v) agarose gel and purified using the Ez-pure PCR purification kit (Ver 2, Enzynomics Co. Ltd., Daejeon, Korea). XhoI and BamHI were used as restriction enzymes and then ligated into pET-21a(+) that had already been digested identically. The recombinant plasmid-carrying *Escherichia coli* BL21 (DE3) was inoculated into LB-ampicillin media and cultured for a whole night at 150 rpm and 37 °C. Recombinant lipase was produced by adding IPTG at a final concentration of 1 mM when the OD 600 reached around 0.4–0.6. After IPTG addition, the temperature was reduced to 16 °C to promote the desired protein expression (Kanjanavas et al. 2010; Zhao et al. 2022). After centrifugation for 15 min at 4 °C and at 7000 rpm, the collected cells were resuspended in a solution containing 50 mM Tris-HCl (pH 8), 200 mM NaCl, and 20 mM imidazole. They were then sonicated five times at 50-amplitude microns, with a 5-min break between each sonication, in an ice bath. Then, only the supernatant was obtained through centrifugation (10,000 rpm, 30 min, 4 °C). Using Ni-NTA column chromatography (QIAGEN) on a column equilibrated with 50 mM Tris-HCl, pH 8, 200 mM NaCl, and 20 mM imidazole at 0.5 mL/min, his-tagged lipase was isolated. The bound protein was eluted 50 mM Tris-HCl (pH 8) and 200 mM NaCl added over a 50–1000 mM imidazole gradient (Kanjanavas et al. 2010). Utilizing 12% SDS-PAGE (Bio-Rad), the purity and molecular weight of the isolated lipase were confirmed. Thermo Fisher Scientific (Waltham, MA, USA) provided a bicinchoninic acid protein assay kit, which was used to measure the protein concentration (mg/mL).

### Lipase activity assay using chromogenic plates

A modified Singh et al. approach was used to measure the enzyme activity (Singh et al. 2006). In brief, 2% agar, 0.1% CaCl_2_, 0.01% phenol red, and 1% olive oil as a lipidic substrate were used to make chromogenic substrate plates. After raising the pH to 7.4 with 0.1 N NaOH, the mixtures were autoclaved at 121 °C for 15 min to accomplish sterilization. The purified lipase solution at 1-, 2-, 10-, 20-, and 50-fold dilutions was dropped onto a filter disc paper overlaid on the prepared chromogenic plates. And then, they were incubated at 37 °C for 1 h before visual inspection of clearing zones as the indicator of lipase activity.

### Sequence and structure analysis

GenBank accession number of the Lac-Rh gene is CP045531.1. Lac-Rh contains 212 amino acids and shares a 75.4% homology with the 281 amino acid NCBI sequence accession number: WP_033573053. The local alignment was performed using BlastP. The secondary structure elements at the sequence level were predicted by the JPred 4 server (Drozdetskiy et al. 2015). The predicted structure of Lac-Rh was predicted using ColabFold in MMseqs2 (Mirdita et al. 2022). Structural visualization and alignments were performed with PyMOLv2.5.

### Lipase activity assay

The lipase assay was carried out according to the method previously described by Gupta et al. (2002). In short, 2 mL of Triton X-100, 100 mg of gum Arabic, and 207 mg of sodium deoxycholate were added to 100 mL of 50 mM potassium phosphate buffer (pH 8). Para-nitrophenyl palmitate (p-NPP) was dissolved in isopropanol and mixed with buffer to a final substrate concentration of 1 mM in order to create the reaction mixture. The assay mixture comprised 100 µL enzyme (44.2 µg) and 100 µL of reaction mixture. Based on this, the reaction was carried out by maintaining the amount of enzyme in experiments under various conditions. The mixture was incubated for 30 min along with a control (without enzyme). The activity was determined by measuring the release of p*-*NP from p*-*NPP at 410 nm. One unit (U) of lipase activity was defined as the amount of lipase liberated 1 µmol p*-*NP from p*-*NPP per min.

### Biochemical characterization of lipase

#### Temperature screen of Lac-Rh activity and stability

To evaluate the activity of Lac-Rh according to temperature, the analysis was performed in the temperature range of 10 to 80 °C, which is commonly found in other enzyme activity papers (Li et al. 2014; Barman and Dkhar 2022), based on the method described in the “Lipase activity assay” section. Lac-Rh was pre-incubated in the buffer at different temperatures (40 °C, 60 °C, and 70 °C) in order to measure the thermostability during a 240-min period. The thermostability was then calculated by measuring the residual activities samples every 30 min (Zhao et al. 2021).

#### pH screen of Lac-Rh activity and stability

The pH of the reaction mixture was adjusted using different buffers in order to evaluate the stability and activity of the Lac-Rh at different pH levels. Activity was tested using different pH buffers including sodium acetate buffer (pH 5–6), potassium phosphate buffer (pH 6–7), Tris-HCl (pH 7–9), and sodium carbonate buffer (pH 9–11). The substrate was produced with the appropriate buffer at a certain pH for the activity test. For the stability test, the Lac-Rh was preincubated with different buffers for 1 h before quantification (Maharana and Ray 2015).

#### Metal ion screen of Lac-Rh activity

To investigate the effect of metal ions on Lac-Rh, metal ions at a final concentration of 1 mM were added to the reaction mixture and lipase activity assays were measured. The experiment was conducted using the optimal conditions determined. The types of metal ions used are Mg^2+^, Mn^2+^, Z^2+^, Ca^2+^, Cu^2+^, and Fe^2+^. The relative activity of the enzyme was calculated as a percentage of that of control, which had no metal ions, and hence was 100%.

#### Substrate specificity assay

To investigate the substrate specificity of Lac-Rh, a reaction solution was prepared by dissolving various p-NP ester derivatives, including caprylate, laurate, palmitate, and stearate, in buffer as described in the “Lipase activity assay” section. Then it was incubated with Lac-Rh for 30 min.

#### Effects of organic solvents on Lac-Rh activity

Aliquots of the purified Lac-Rh (44.2 µg) were incubated with polar organic solvents including acetone, glycerol, DMSO, methanol, ethanol, acetonitrile, isopropyl alcohol, ethyl acetate, hexane, and n-butyl acetate at 10% and 30% (v/v) in Tris-HCl buffer (pH 8) for 1 h in order to examine the impact of organic solvents on lipase activity. The residual lipase activity was measured after incubation at 60 °C for 1 h.

#### Effects of commercial detergents on Lac-Rh activity

To investigate the feasibility of using the purified Lac-Rh as a detergent additive, the enzyme activity was investigated using four commercially available detergents (Tamsa, Sugar bubble, Dawny, and Persil). The endogenous enzyme in each detergent was made inactive by autoclaving the diluted detergents at 121 °C for 15 min following diluting each detergent with distilled water to a ratio of 10% (v/v). Detergent solutions were again diluted to final concentrations of 1% and 5% before the adding 44.2 µg of the purified Lac-Rh (Zhao et al. 2021; Grbavčić et al. 2011; Abol-Fotouh et al. 2021; Cherif et al. 2011). The mixtures were maintained at 25 °C for 2 h (Abol-Fotouh et al. 2021). Residual activity was calculated as 100% with respect to testing without preincubation in detergent. A sample treated only with inactivation of the endogenous enzyme was placed as a negative control.

#### Evaluation of Lac-Rh for detergent performance

Lac-Rh’s efficacy as an ingredient in laundry detergent was assessed using cotton cloth (5 × 5 cm). Cotton fabric pieces were first cleaned in boiling chloroform for 4 h to remove any potential oils, and then left to air dry at room temperature for the whole night (Sharma et al. 2017; Grbavčić et al. 2011; Li et al. 2014). Defatted cloths were stained with chocolate, olive oil, and peanut oil. For the oils, a purple fat-soluble pigment (liquid candy color violet, Chefmaster) was added to make the stain easier to see with the naked eye. The dyed fabrics were dried at room temperature for 1 h (Sharma et al. 2017; Grbavčić et al. 2011). For washing performance, three different compositions of washing solution were prepared as listed below (a–c) (Sharma et al. 2017; Gurkok and Ozdal 2021; Zafar et al. 2022; Maharana and Ray 2015; Nimkande and Bafana 2022). The cotton fabric was washed by soaking it in various washing solutions for 1 h at 50 °C while being shaken at 150 rpm. It was then rinsed in distilled water and allowed to dry at room temperature. Digital camera images of the textiles were taken (Sharma et al. 2017; Abol-Fotouh et al. 2021).

-  100 mL of distilled water + stained cloth.
-  100 mL of 1% (v/v) heat-inactivated detergent solution + stained cloth.
-  100 mL of 1% (v/v) heat-inactivated detergent solution + 2 mL enzyme solution (442.4 µg/mL) + stained cloth.

### Statistical analysis

The mean ± standard deviation of three separate experiments is exhibited. Using a one-way analysis of variance, significant differences were found. A statistically significant difference was defined as having a probability value of *p* < 0.05.

## Results

### Isolation and activity detection on agar plate

**Fig. 1:**
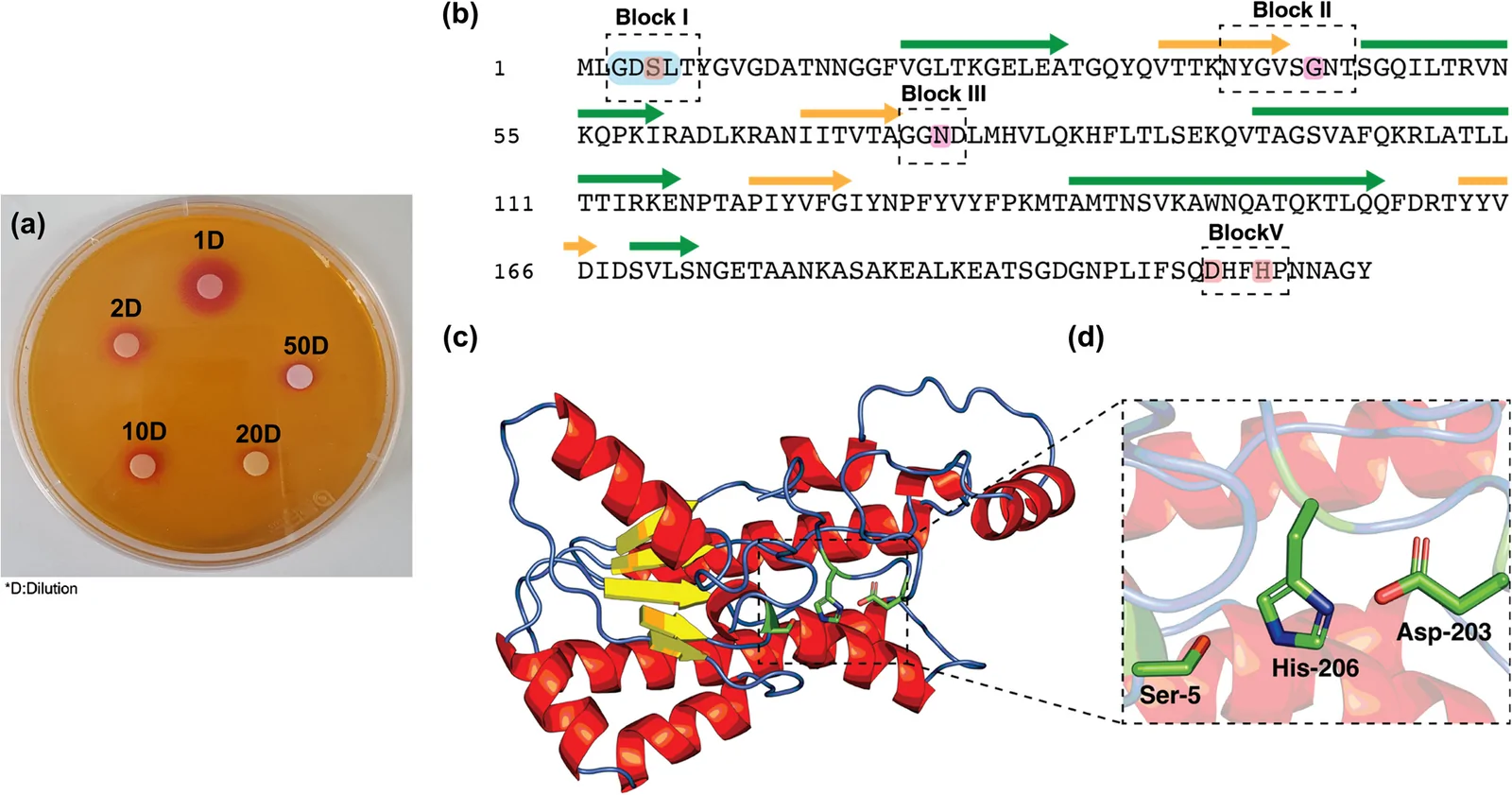
(**a**) Detection of lipase from *L. rhamnosus* IDCC 3201 on agar plates containing olive oil at 1-, 2-, 10-, 20-, and 50-fold dilutions of the purified lipase solution (dilution is denoted by D). (**b**) Amino acid sequence of Lac Rh. The GDSL motif is highlighted in the shaded blue box, the catalytic residues in red, and conserved sequence blocks of a GDSL family member I, II, III, and V in dashed black boxes. The catalytic triad and oxyanion hole residues are indicated by red and pink shaded boxes, respectively. The secondary structure annotation of sequence is shown, with green arrows to represent helices and orange arrows to denote strands as predicted by the JPred 4 server. Lac-Rh contains 212 amino acids and shares a 75.4% homology with the 281 amino acid NCBI sequence accession number: WP_033573053. (**c**) Lac-Rh shares structural features common to the GDSL hydrolase subfamily of lipolytic enzymes. The Colabfold structure reveals an active site common to the GDSL subfamily. The cartoon representation shows the key secondary structure elements and the active site. (**d**) The catalytic triad of Lac-Rh (Asp-203, His-206, and Ser-5) are colored in green and shown as sticks

Lac-Rh is a novel candidate enzyme from *L. rhamnosus* IDCC 3201 that was isolated and subsequently successfully expressed and purified in *Escherichia coli*. A single band at about 24 kDa indicated successful purification (Supporting Information Fig. S1). The protein concentration of purified eluate from a 1-L culture was 442.5 µg/mL. Applying the standard chromogenic test, the isolated enzyme’s activity was qualitatively evaluated (Fig. 1). A yellow zone was observed only in regions where Lac-Rh was impregnated on the disc. After a 1-h incubation at 37 °C on a plate containing olive oil as a substrate, a clear hydrolysis region appeared (Fig. 1a), which increased in diameter with increasing enzyme loading.

### Amino acid sequence and structural analyses

The analysis of the amino acid sequence of Lac-Rh confirmed a G-D-S-L motif between amino acid-residues 3 and 6 close to the N-terminus, a motif typical of the SGNH/GDSL hydrolase superfamily (Akoh et al. 2004; Chepyshko et al. 2012) (Fig. 1b). Subsequent BLASTP analysis using the non-redundant protein database in NCBI revealed that Lac-Rh showed similarity to several enzymes including the SGNH/GDSL hydrolase family protein (NCBI sequence accession numbers: WP_033573053.1, WP_005713864.1, WP_0.005686405.1, WP_101904007.1, WP_204148790.1) from *L. rhamnosus* (identity 75.4%), hypothetical protein N507_0129 (ASY47324.1) from *L. rhamnosus* DSM 14870 identity 74.4%), GDSL-like protein (EHJ34214.1) from *L. rhamnosus* ATCC 21052 (identity 74.4%), lipase (HAJ56010.1) from *Lactiplantibacillus* sp. (identity 77%), and lipase (KMO45285.1) from *L. rhamnosus* (identity 87.9%). The BLASTP analysis indicated moderate similarity (≤ 50%) with the lipase (QFQ91291.1) from *L. manihotivorans*, and the GDSL-type esterase/lipase family protein (WP_204118710.1) from *L. suilingensis* (identitiy 50.1%) and from *Lactiplantibacillus* sp. (identitiy 49.8%). The common feature of all enzymes homologous to Lac-Rh is that they belong to the SGNH-hydrolase superfamily known as the “SGNH-hydrolase YpmR like (cd0406)” subfamily. Their tertiary fold differs significantly from the α/β hydrolase family, with an active site closely resembling the typical Ser-His-Asp (or Glu) triad of other serine hydrolases but potentially lacking a carboxylic acid (Yu et al. 2016; Wang et al. 2023). Lac-Rh was not classified as a conventional α/β lipase as it lacked the highly conserved pentapeptide G-x-S-x-G motif.

The GDSL-hydrolases are a diverse family of serine lipases and esterases found throughout all kingdoms of life (Akoh et al. 2004; Chepyshko et al. 2012) and are one of four families that comprise the wider SGNH superfamily. Members of the GDSL family are categorized based on the existence of four highly conserved homology-rich conserved sequence blocks (I–IV) (Akoh et al. 2004; Chepyshko et al. 2012). The four homology blocks were confirmed in Lac-Rh sequence and are shown labeled in Fig. 1b. Block I contains the GDSX motif and the nucleophilic Ser residues while the consensus amino acids Gly, Asn, and His are found in blocks II, III, and V respectively (Akoh et al. 2004; Chepyshko et al. 2012; Mirdita et al. 2022). In Lac-Rh, these correspond to residues Ser-5, Gly-44, Asn-77, and His-206. An additional feature for block V is a catalytic Asp located at the third amino acid preceding the catalytic histidine (DxxH), a sequence feature also seen in Lac-Rh.

The structure of Lac-Rh was predicted using Colabfold. Lac-Rh shares a typical fold of the SGNH-hydrolase superfamily, comprising a central five-stranded parallel β-sheet surrounded by α-helices (Fig. 1c). The active site comprises the catalytic triad Ser-His-Asp common to other serine hydrolases (Fig. 1d), although the tertiary fold of the enzyme is substantially different from that of common α/β hydrolase lipases and does not contain the nucleophilic elbow.

### Effects of temperature and pH on activity and stability

**Fig. 2:**
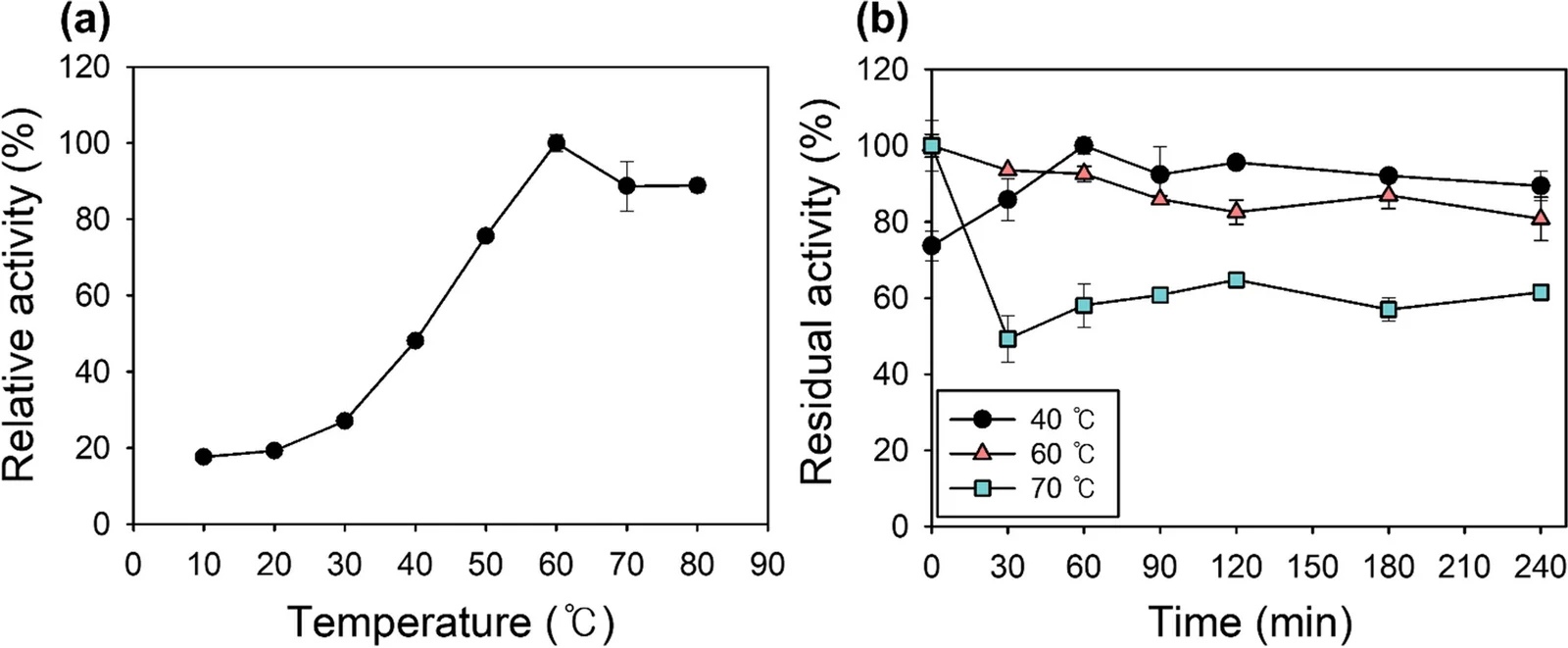
Effect of temperature on optimum activity and stability. (**a**) Optimum temperature of Lac-Rh was determined from 10 to 80 °C. The activity assay was performed in 50 mM potassium phosphate buffer, pH 8.0. (**b**) To determine the thermal stability, the purified lipase was incubated at 40 °C, 60 °C, and 70 °C for 240 min

**Fig. 3:**
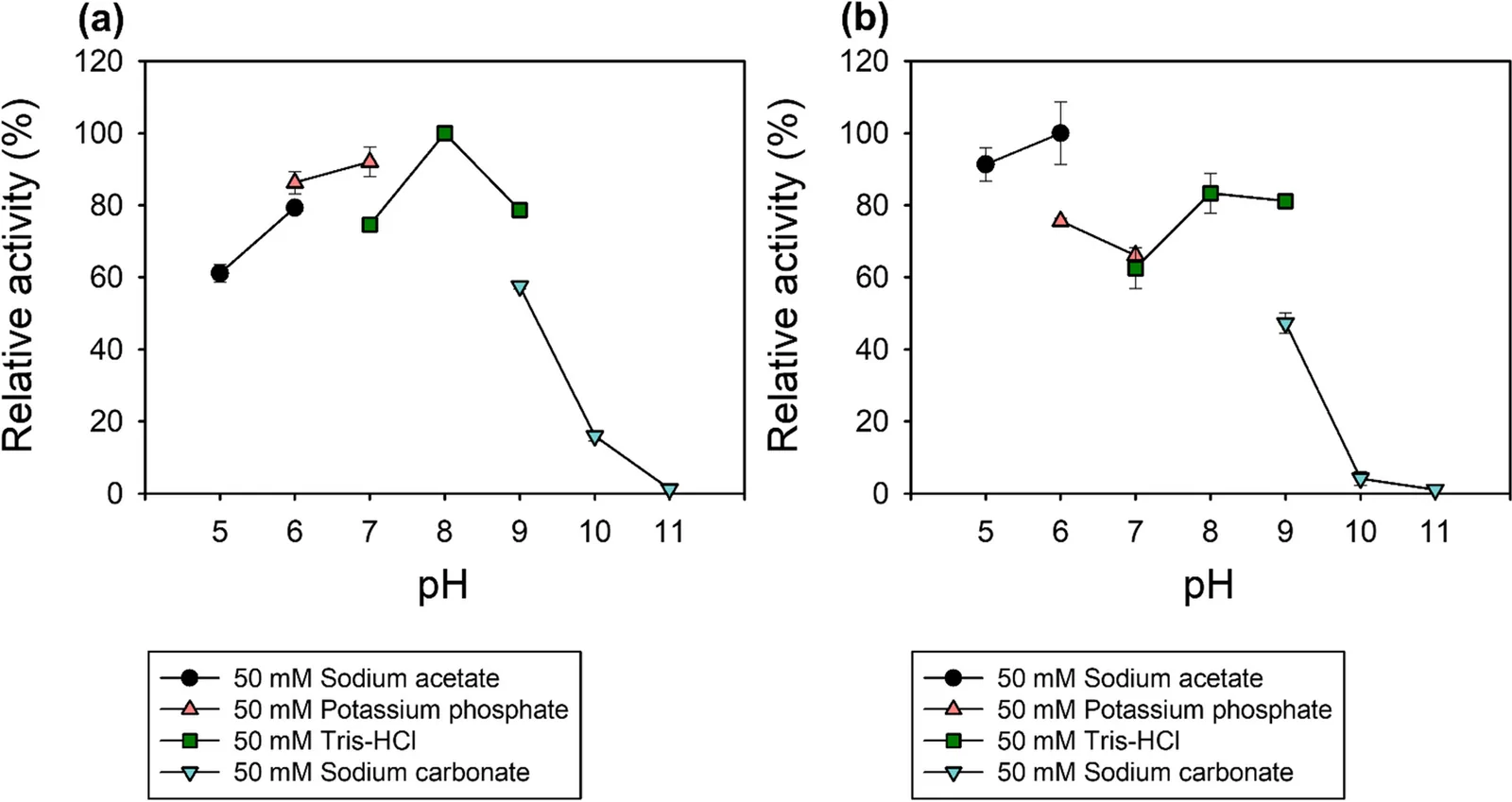
Effect of pH on optimum activity and stability of Lac-Rh. (**a**) The optimum pH for lipase activity was investigated at 60 °C. Buffers used for the assays were 50 mM sodium acetate buffer (pH 5–6), 50 mM potassium phosphate buffer (pH 6–7), 50 mM Tris-HCl buffer (pH 7–9), and 50 mM sodium carbonate buffer (pH 9–11). (**b**) To determine pH stability, the purified lipase was incubated in different buffers (pH 5–11) at 60 °C for 1 h

Most industrial enzymatic processes operate at 50 °C or above and therefore, stable enzyme activity at high temperature is a key requirement (Lailaja and Chandrasekaran 2013). A temperature range of 0 to 80 °C was used to measure Lac-Rh activity, and the maximum lipase activity (3438.5 ± 1.8 U/mg) was shown at 60 °C. The Lac-Rh activity gradually increased up to 60 °C and was retained at 89% until 80 °C (Fig. 2a). To investigate the thermal stability over time, the enzyme was incubated at 40 °C, 60 °C, and 70 °C for 4 h. After incubation for 4 h, the Lac-Rh retained 80% of initial activity at 40 °C and 60 °C (Fig. 2b). However, at 70 °C, the residual activity decreased to 60%. Furthermore, the effect of pH on the lipase activity of Lac-Rh was determined across a pH range of 5 to 11 (Fig. 3a) where its optimum activity was established to be at pH 8 in 50 mM Tris-HCl (Fig. 3a). The residual Lac-Rh activity was after incubation at 60 °C for 1 h from pH 5 to pH 11 (Fig. 3b) retained 80% and 90% of the initial activity at pH 5–6 and 8–9, respectively. No activity was detectable at pH 10 or 11.

### Effects of various metal ion on activity

The influence of metal ions on lipase activity was determined by incubation with concentrations of 1 mM of a range of metal ions (Table 1). Lac-Rh activity did not increase in the presence of any of the metal ions, suggesting that metal ions were not required as cofactors (Khan et al. 2023). Notably, the residual lipase activity was reduced to 90.8 ± 3.2% in the presence of Ca^2+^.

**Table 1:** Effect of various metal ions on lipase activity. Lipase activity assay was performed in 50 mM Tris-HCl buffer (pH 8.0) with addition of 1 mM of each metal ion. Activity of control (no metal ions in the mixtures) is 100%

Metal ions	Relative activity (%)
Control	100.00 ± 0.01^bcd^
MgCl_2_	104.5 ± 0.7^cd^
MnCl_2_	99.3 ± 0.2^bc^
ZnCl_2_	96.5 ± 0.4^b^
CaCl_2_	90.8 ± 3.2^a^
CuCl_2_	104.6 ± 4.0^d^
FeCl_2_	102.2 ± 1.0^cd^

^a–d^Means with different letters within a same column are significantly different at *p* < 0.05

### Substrate specificity against pNP-esters

Lac-Rh demonstrated activity against C10–C18 para-nitrophenyl (pNP) esters, indicating its preference for long-chain esters (Table 2). The highest enzyme activity was seen for the p-nitrophenyl laurate (p-NPL) ester containing 12 carbons. This is consistent with studies on other microbial lipases, confirming a preference toward pNP ester derivatives with > 10 carbon atoms (Zhao et al. 2021, 2022; Akmoussi-Toumi et al. 2018).

**Table 2:** Substrate specificity of enzyme against C10-C18 esters, activity measured in 50 mM Tris-HCl buffer (pH 8.0)

Substrate	Relative activity (%)
p-NP caprate (C10)	70.7 ± 2.2^a^
p-NP laurate (C12)	100.0 ± 3.7^b^
p-NP palmitate (C16)	95.1 ± 5.9^b^
p-NP stearate (C18)	71.7 ± 5.7^a^

^a–d^Means with different letters within a same column are significantly different at *p* < 0.05

### Effects of organic solvents on activity

**Fig. 4:**
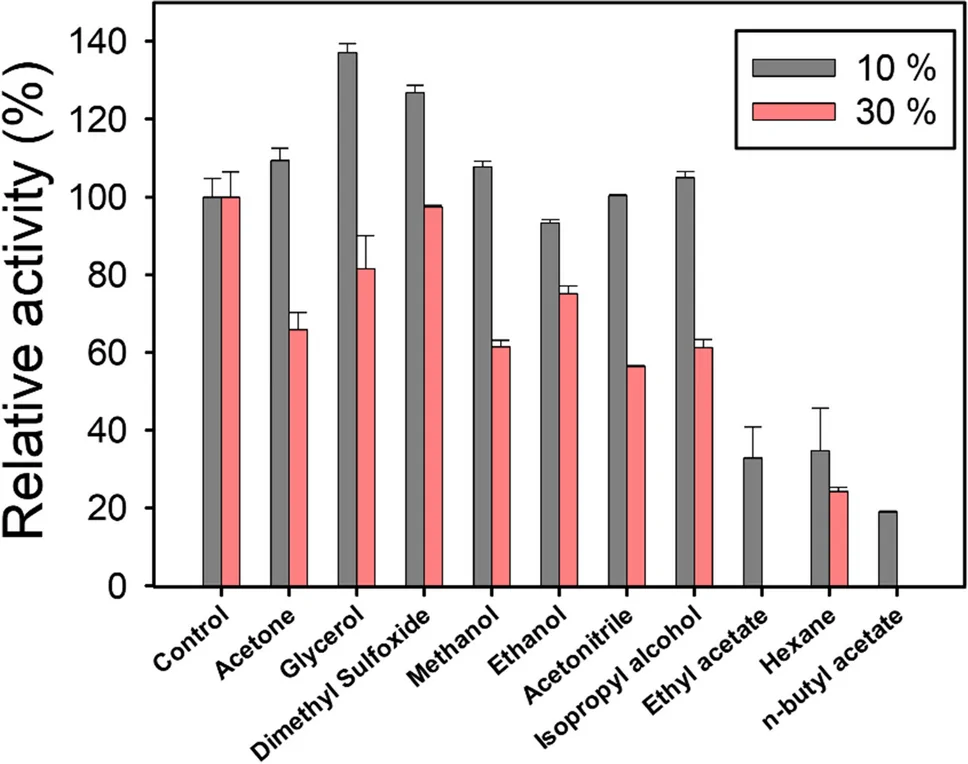
Influence of organic solvents at different concentrations (10% and 30%) on the activity of Lac-Rh. The activity of Lac-Rh was estimated after preincubation with organic solvents at room temperature for 1 h. The activity of control (no organic solvents in the mixtures) is 100%

Lipases are generally known as biocatalysts which exhibit high tolerance to organic solvents for detergent, biotransformation, and organic synthesis applications (Chakraborty and Raj 2008). The solvent tolerability of Lac-Rh was evaluated by preincubating the enzyme for 1 h in both 10% and 30% (v/v) organic solvents followed by determination of the enzyme activity (Fig. 4). Interestingly, the activity of Lac-Rh was increased in acetone, glycerol, dimethyl sulfoxide, and methanol at 10% (v/v), whereas neither acetonitrile nor isopropyl alcohol had any influence on enzyme activity compared with that of the control. The enzyme’s maximal relative activity in 10% (v/v) glycerol was found to be 137.1 ± 2.4%. An increase in activity was also observed in dimethyl sulfoxide (DMSO), methanol, and isopropyl alcohol to 126.8 ± 2.0%, 107.7 ± 1.6%, and 105.0 ± 1.6%, respectively. However, a loss in relative lipase activity was observed when the concentration of all organic solvents was increased to 30% (v/v) (Fig. 4). The loss of activity was evident in most organic solvents, except for glycerol, DMSO, and ethanol (81.6 ± 8.4%, 97.3 ± 0.5%, and 75.2 ± 2.0% residual activity, respectively) while other solvents resulted in < 70% activity. Activity was completely lost in 30% ethyl acetate and n-butyl acetate.

#### Effects of commercial detergents on activity

**Fig. 5:**
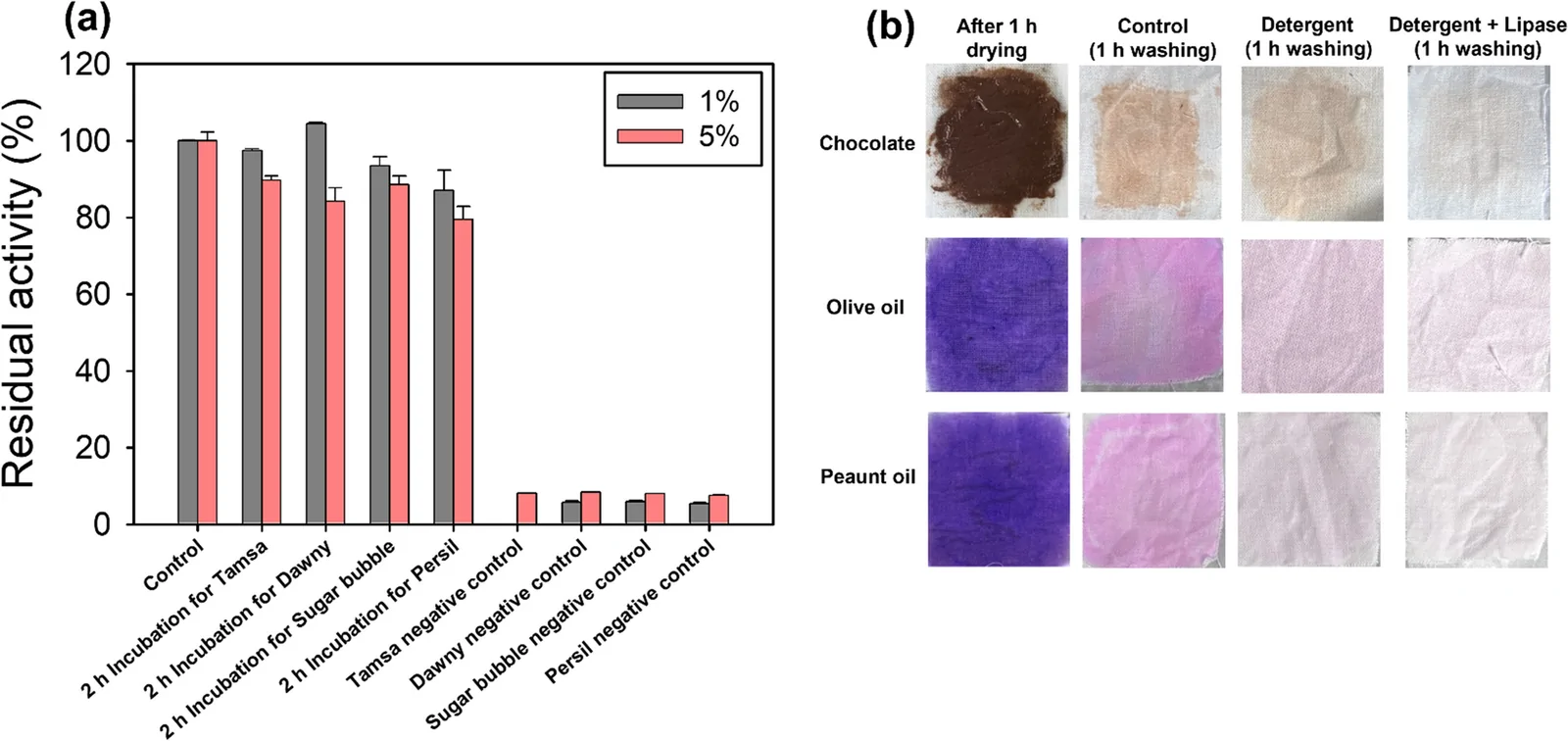
Detergency of Lac-Rh. (**a**) Influence of commercial detergents at 1% and 5% concentrations on the Lac-RH activity estimated after incubation with detergents at room temperature for 2 h. (**b**) Lac-Rh was applied to a heat-deactivated detergent to study the efficacy of the removal of different stains (chocolate, olive oil, and peanut oil) from cotton cloth. Control is the cotton cloth treated with distilled water during 1 h. Detergent is the effect of heat-inactivated detergent on the stains during 1 h. Detergent + Lipase is the heat-inactivated detergent supplemented with the Lac-Rh on the stains during 1 h

To investigate the potential application of the Lac-Rh as a detergent additive, the activity was evaluated with various commercial detergents, including Tamsa, Sugar bubble, Dawny, and Persil (Grbavčić et al. 2011) at 1% and 5% (Fig. 5a). The negative control, an inactivated endogenous enzyme in the commercial detergent, showed zero activity. Importantly, Lac-Rh preincubated at both 1% and 5% v/v of the commercial detergent showed a residual activity of ≥ 80% (Fig. 5a). Thus, the lipase had considerable detergent compatibility, confirming its potential as a detergent additive (Zhao et al. 2021; Chauhan et al. 2013; Saraswat et al. 2017; Grbavčić et al. 2011; Akmoussi-Toumi et al. 2018; Abol-Fotouh et al. 2021; Gurkok and Ozdal 2021).

### Evaluation of the washing performance

Today, laundry is mainly performed using detergents to remove oil stains derived from household industrial sources (Hemlata et al. 2016; Al-Ghanayem and Joseph 2020). Most commercial detergents are hydrolytic enzyme-based detergents that are diluted with water before use (Cherif et al. 2011). Therefore, the stain removal capability of Lac-Rh was examined by incorporating the purified lipase into a diluted detergent solution mixed with water to stimulate modern laundry conditions. Chocolate and two food oils were applied onto swatches of cloths and dried to create stains before enzymatic treatment (Fig. 5b). A control treatment with water alone had little to no effect on stain removal. Treatment with water combined with detergent improved stain removal relative to the control but did not completely remove stains. However, treatment of the strains with Lac-Rh with both water and detergent effectively achieved stain removal, and the white color of the original cloth was observed (Fig. 5b).

## Discussion

Enzymes, particularly hydrolases like lipases, carbohydrases, and proteases, are essential for various industrial processes and are predicted to become a $10.7 billion industry in 2024 (Ramnath et al. 2017). Lipases have risen in prominence as biocatalysts, constituting nearly 10% of the enzyme market, and contribute significantly to a multibillion-dollar bioindustry (Khan et al. 2023). Microbial lipase sources are ideal for industrial use because of their fast growth, genetic stability, and genetic versatility (Chandra et al. 2020). However, to date, a relatively small portion (< 10%) of the known bacterial lipolytic enzymes have been cloned, expressed, and experimentally studied (Kovacic et al. 2019). In this study, a lipase was obtained from the lactic acid bacterium *L. rhamnosus* IDCC 3201 through cloning and purification, and then the lipolytic activity of Lac-Rh was investigated under various conditions to demonstrate the applicability of Lac-Rh for use in the detergent industry.

Lac-Rh can be classified as a SGNH/GDSL hydrolase as it contains the conserved GDS(L) amino acids including the active site Ser, located near the N-terminus (Akoh et al. 2004; Escuder-Rodríguez et al. 2022) (Fig. 1). The block V of a GDSL hydrolase has a flexible active site where Asp is located three amino acids before the His in the catalytic triad, which allows these enzymes to acts as esterases and proteases (Akoh et al. 2004; Chepyshko et al. 2012). This motif offers a distinct catalytic mechanism compared with traditional GxSxG motif hydrolases, and notably, lacks a nucleophilic elbow and features a dynamic active site (Akoh et al. 2004; Chepyshko et al. 2012; Castilla et al. 2022; Román Naranjo et al. 2020). The catalytic Ser is situated within the GDS(L) motif, with Gly and Asn acting as proton donors for the oxyanion hole, whereas His enhances the nucleophilicity of the Ser by removing the proton from its hydroxyl group (Privé et al. 2013). The oxyanion hole, along with the catalytic residues, crucially stabilizes transition states (Oh et al. 2019). The sequence of Lac-Rh possesses shared sequence motifs from family V bacterial GDSL lipases, which indicates that it can be classified within this family, even though it has a smaller, single-domain structure with a molecular mass of approximately 23 kDa (Supporting information), which differs from the typical 30-kDa range of family V lipases (Chandra et al. 2020; Privé et al. 2013).

To screen the enzymatic properties of Lac-Rh, various conditions such as temperature, pH, and metal ions were screened for their effect on activity. Industrial biocatalysts require strong thermostability as temperature changes can considerably affect enzymatic reactions (Tang et al. 2017). Lac-Rh maintained 60% of residual activity at 70 °C even after 4 h (Fig. 2) and exhibited excellent stability against high temperatures. Therefore, the high thermostability of Lac-Rh provides a competitive advantage in industrial enzyme reactions. Most known lipases have an optimum pH between pH 7 and 8 (Lailaja and Chandrasekaran 2013; Gao et al. 2018), whereas the optimal range of activity for lipases used in detergents is generally between pH 8 and 12, and Lac-Rh meets this criterion (Fig. 3) (Vivek et al. 2022).

In terms of substrate specificity, Lac-Rh showed activity against a natural lipid, olive oil (Fig. 1), in addition to pNP substrates with > 10 carbons (Table 2). Oil stains that occur in daily life are usually composed of various long-chain fatty acid esters (Li et al. 2014). Therefore, these results demonstrate that Lac-Rh may be effective against oil stains. Several investigations have shown that the presence of metal ions either increases or decreases lipase activity (Rmili et al. 2019; Sharma et al. 2017; Tang et al. 2017; Zhao et al. 2021; Zarinviarsagh et al. 2017; Akmoussi-Toumi et al. 2018; Gurkok and Ozdal 2021; Ben Bacha et al. 2018). In this study, the activity of lLac-Rh remained unaffected whether metal ions were present or not, although there was a little reduction in the presence of calcium ions (Table 1). It is probably because the thiol groups of Cys residues near the enzyme’s active region may have interacted with the ions (Dab et al. 2023).

While eukaryotes, including plants, are known to possess many GDSL hydrolases involved in defense and metabolism, the isolation and characterization of bacterial SGNH hydrolases are limited (Touray et al. 2020; Jo et al. 2021). Although the diversity of microbial lipases may provide varying sensitivities to different organic solvents, several previous studies have shown that bacterial enzymes containing the GDSL motif generally exhibit resistance to metals, organic solvents, and detergents (Ding et al. 2014; Hemlata et al. 2016; Li et al. 2020; He et al. 2024). The characteristics of resistance to a variety of extreme conditions derived from the unique structure of the SGNH/GDSL hydrolase make it a highly desirable biocatalyst for applications encompassing food processing, flavors and perfumes, cosmetics, pharmaceuticals, and laundry detergents (Hong et al. 2019). Typically, water-miscible polar solvents tend to be more destabilizing to lipase stability than nonpolar solvents, and lipase stability is reported to benefit from the use of nonpolar hydrophobic solvents (Hemlata et al. 2016; Zhao et al. 2021; Mander et al. 2012). However, in our study, Lac-Rh showed increased activity in 10% (v/v) glycerol, DMSO, acetonitrile, methanol, and ethanol (Fig. 4). Lac-Rh notably exhibited the highest activity in highly polar organic solvent glycerol, achieving a relative activity level of 137.1 ± 2.4% at a 10% (v/v) glycerol concentration. This phenomenon may be attributed to glycerol being a byproduct of triacylglycerol hydrolysis, suggesting that Lac-Rh could be a promising candidate for enzymatic hydrolysis with minimal product inhibition (Zhao et al. 2021; Chakraborty and Raj 2008). Increased activity with glycerol addition has also been observed with other microbial lipases from *Streptomyces* (Mander et al. 2012; Zhao et al. 2022). Finally, a significant negative effect on Lac-Rh activity was measured in ethyl acetate, hexane, and n-butyl acetate solvents, which is likely due to a rapid structural denaturation or disruption of the active site (Hemlata et al. 2016; Akmoussi-Toumi et al. 2018; Zhao et al. 2022).

Detergents with enzymes offer superior cleaning performance and also provide important ecological benefits by reducing the amount of detergent required (Khan et al. 2023; Gurkok and Ozdal 2021; Castilla et al. 2022; Vivek et al. 2022). Lipolytic enzymes are specifically added to detergents for their grease-removing capabilities for various stains under typical washing conditions (Castilla et al. 2022). Lipases suitable for the laundry detergent industry must remain stable over a wide range of temperatures and alkaline pH conditions (Dab et al. 2023) and have compatibility with commonly used surfactants and oxidizing agents (Dab et al. 2023; Abol-Fotouh et al. 2021; Li et al. 2014). Lac-Rh successfully maintained stable activity in four different commercial detergents and effectively removed oily stains such as chocolate, olive oil, and peanut oil. Therefore, under intensive operational washing conditions, such as in a washing machine, Lac-Rh can be expected to completely remove oily stains on clothing (Dab et al. 2023). Collectively, our study indicates the potential of Lac-Rh in the development of novel detergent formulations.

In conclusion, a novel lipase gene isolated from *L*. *rhamnosus* IDCC 3201 strain was cloned, expressed, purified, and characterized. The purified Lac-Rh showed high thermal and pH stability as well as resistance to a variety of organic solvents. Moreover, Lac-Rh demonstrated excellent stability in commercial detergents and improved the detergent’s capacity to eliminate oil stains from fabrics. Overall, these results demonstrate that a novel lipase from *L*. *rhamnosus* IDCC 3201 has potential as an auxiliary ingredient for the production of detergents.

## Supplementary Information

Below is the link to the electronic supplementary material.

Supplementary file1 (PDF 290 KB)

## Data Availability

The study’s supporting data can be found in the main manuscript and Supplementary Information. For access to the raw data, please reach out to the corresponding author with a reasonable request.
