# Supplementary material for: A lipase from Lacticaseibacillus rhamnosus IDCC 3201 with thermostability and pH resistance for use as a detergent additive
Source: Appl Microbiol Biotechnol. 2024 Jun 6;108(1):365. doi: 10.1007/s00253-024-13185-4 (PMC11156721; doi:10.1007/s00253-024-13185-4)
Supplement: Supplementary file 1 — Supplementary file1 (PDF 290 KB) [file 253_2024_13185_MOESM1_ESM.pdf]

Applied Microbiology and Biotechnology

A lipase from *Lacticaseibacillus rhamnosus* IDCC 3201 with thermostability and pH resistance for use as a detergent additive

Running title: A thermostable lipase from *Lacticaseibacillus rhamnosus* IDCC 3501

Mi Dan Kang[1,†], Go Eun Choi[1,†] Jeong Hwa Jang[1,†], Sung-Chul Hong[2], Hee-Soo Park[1], Dong Hyun Kim[1], Won Chan Kim[3], Natasha P. Murphy[4*], Young Hoon Jung[1*]

[1]School of Food Science and Biotechnology, Kyungpook National University, Daegu 41566, Republic of Korea

[2] Department of Food Science and Biotechnology, Kunsan National University, Gunsan 54150, Republic of Korea

[3] Department of Applied Biosciences, Department of Integrative Biology, Kyungpook National University, Daegu, 41566, Republic of Korea

[4]Renewable Resources and Enabling Sciences Center, National Renewable Energy Laboratory, Golden, Colorado 80401, United States

[†]These authors equally contributed to this study.

* Correspondence:

Young Hoon Jung; Email: younghoonjung@knu.ac.kr; Tel: +82-53-950-5777; Fax: +82-53-950-6777

Natasha P. Murphy; Natasha.murphy@nrel.gov; Tel: +1-303-630-5226; Fax: +1-303-

630-5226

This PDF file includes Supplementary Figure S1

Fig. S1

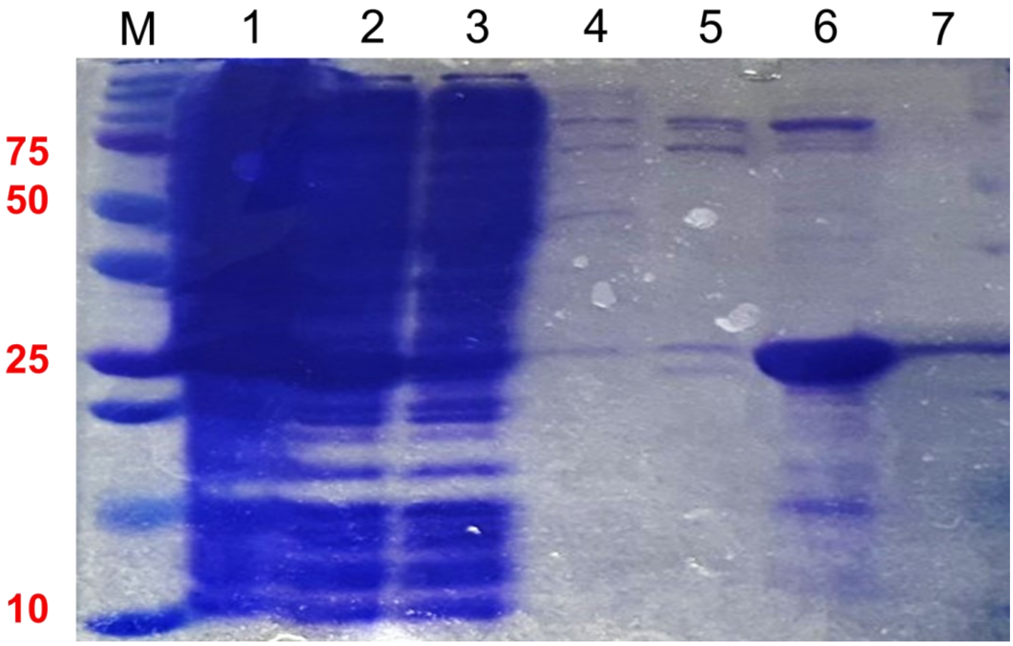

Fig S1. SDS-PAGE analysis of purification of lipase from *L. rhamnosus* IDCC 3201 with different imidazole concentrations. Lane M: Protein marker; lane 1: pellet; lane 2: crude; lane 3: flow through; lane 4: 20 mM imidazole; lane 5: 50 mM imidazole; lane 6; 300 mM imidazole; lane 7; 1 M imidazole.
